# Electroacupuncture to treat gastroesophageal reflux disease: study protocol for a randomized controlled trial

**DOI:** 10.1186/s13063-016-1371-8

**Published:** 2016-05-17

**Authors:** Gajin Han, Jungtae Leem, Hojung Lee, Junhee Lee

**Affiliations:** Korean Medicine Clinical Trial Center, Kyung Hee University Korean Medicine Hospital, 23 Kyungheedae-ro, Dongdaemun-gu, Seoul, 02447 Republic of Korea; Department of Gastroenterology, College of Korean Medicine, Kyung Hee University, 26 Kyungheedae-ro, Dongdaemun-gu, Seoul, 02447 Republic of Korea; Department of Clinical Korean Medicine, Graduate School, Kyung Hee University, 26 Kyungheedae-ro, Dongdaemun-gu, Seoul, 02447 Republic of Korea; Department of Sasang Constitutional Medicine, College of Korean Medicine, Kyung Hee University, 26 Kyungheedae-ro, Dongdaemun-gu, Seoul, 02447 Republic of Korea

**Keywords:** Gastroesophageal reflux disease, Electroacupuncture, Park device, Proportion of responders, Pattern identification

## Abstract

**Background:**

Gastroesophageal reflux disease lowers the quality of life and increases medical costs. Electroacupuncture has been used to ease symptoms and improve gastrointestinal motility in patients with gastroesophageal reflux disease. The main purposes of this study are to evaluate the efficacy and safety of this procedure.

**Methods/design:**

This is a protocol for a randomized, patient-blinded, assessor-blinded, sham-controlled trial. Sixty participants with symptoms of gastroesophageal reflux disease, who have previously undergone standard treatment, will be recruited from August 2015 at Kyung Hee University Korean Medicine Hospital. The participants will be allocated to either the electroacupuncture (n = 30) or the sham electroacupuncture group (n = 30); the allocation will be concealed from both the participants and the assessors. The EA group will undergo penetrating acupuncture at 18 fixed points and two optional points chosen using the pattern identification for gastroesophageal reflux disease. Electrical stimulation will be applied at some of the acupoints. The sham electroacupuncture group will undergo nonpenetrating acupuncture without electrical stimulation at 18 nonspecific points, each of which will be only 2 cm away from the true acupoints used in the electroacupuncture group. In both groups, the procedure will be performed using the Park device. The treatment will last for 6 weeks (with two sessions each week), and the outcome will be evaluated at baseline, 3 weeks, and 6 weeks. The primary outcome will be the proportion of responders with adequate symptom relief, whereas the secondary outcomes will comprise the results of the Nepean dyspepsia index; the Korean gastrointestinal symptom rating scale; the EQ-5D™; levels of gastrin, motilin, and inflammatory cytokines; the perceived stress scale; the qi-stagnation questionnaire; the patient global impression of change; and the spleen qi deficiency questionnaire.

**Discussion:**

The results of this trial will provide information about the efficacy and safety of electroacupuncture in the treatment of gastroesophageal reflux disease symptoms, as well as evidence regarding the use of electroacupuncture to treat gastroesophageal reflux disease in real clinical practice.

**Trial registration:**

Clinical Research Information Service Identifier, KCT0001653. Registered on 12 October 2015.

**Electronic supplementary material:**

The online version of this article (doi:10.1186/s13063-016-1371-8) contains supplementary material, which is available to authorized users.

## Background

Recently, the prevalence of gastroesophageal reflux disease (GERD) in Asia has been increasing, and GERD has now become an important disease [1–3]. Most patients with GERD require long-term therapy, as it is a chronic condition [4]. The standard treatment for GERD is known as a proton pump inhibitor (PPI), which heals the esophageal mucosa and eases the symptoms of GERD; the PPI has become a mainstay of GERD treatment [5]. However, approximately 20 % to 30 % of GERD patients continue to experience symptoms despite PPI treatment [6].

For this reason, interest is growing in complementary and alternative medicine—including acupuncture [5]. Recently, acupuncture has been reported to possibly ease gastrointestinal symptoms [7] and improve esophageal motility [8]. For instance, according to several previous animal studies [9–11], electroacupuncture (EA) increases abnormally low esophageal sphincter pressure (ESP) and reduces the frequency of transient lower esophageal reflux relaxations (TLESRs); in so doing, the treatment increases esophageal motility. GERD usually develops because of a decrease in ESP and the esophageal clearance capacity, as well as an increase in TLESR frequency [12]. Taken together, EA likely ameliorates GERD by controlling esophageal motility. However, little has been reported regarding the efficacy of EA in GERD; to our knowledge, no randomized controlled trials (RCTs), where a control group undergoes a sham procedure, have been carried out to evaluate the effect of EA on GERD in humans.

On another note, both gastrin and motilin are related to ESP, yet the influence of EA on the levels of these proteins has been disputed in previous studies [11, 13–16]. Therefore, with a view to resolving this controversy, we will evaluate the differences in gastrin and motilin levels after EA. Meanwhile, one report has stated that inflammatory mediators are associated with esophageal motility in patients with GERD [17]. For this reason, before and after treatment, we will test for several inflammatory cytokines present in GERD to evaluate the influence of EA on the levels of these cytokines.

In clinical practice, many patients who continue to suffer symptoms of GERD despite standard treatments turn to traditional Korean medicine—specifically EA. Nonetheless, there remains insufficient evidence from well-designed RCTs to support EA use. Hence, we have designed this trial to evaluate the efficacy and safety of partially individualized EA in patients with GERD who have already undergone standard therapy (PPIs or lifestyle modification). In the trial, an EA group will be compared with a sham EA control group. In addition, we will clarify the influence of EA on gastrointestinal (GI) hormones and inflammatory cytokines in patients with GERD.

## Methods/design

This protocol was prepared according to the Standard Protocol Items: Recommendations for Interventional Trials (SPIRIT) 2013 statement. See Additional file 1 for the completed SPIRIT checklist.

### Hypothesis

Partially individualized EA treatment is superior to sham EA in terms of easing the symptoms of GERD.

### Design

**Fig. 1:**
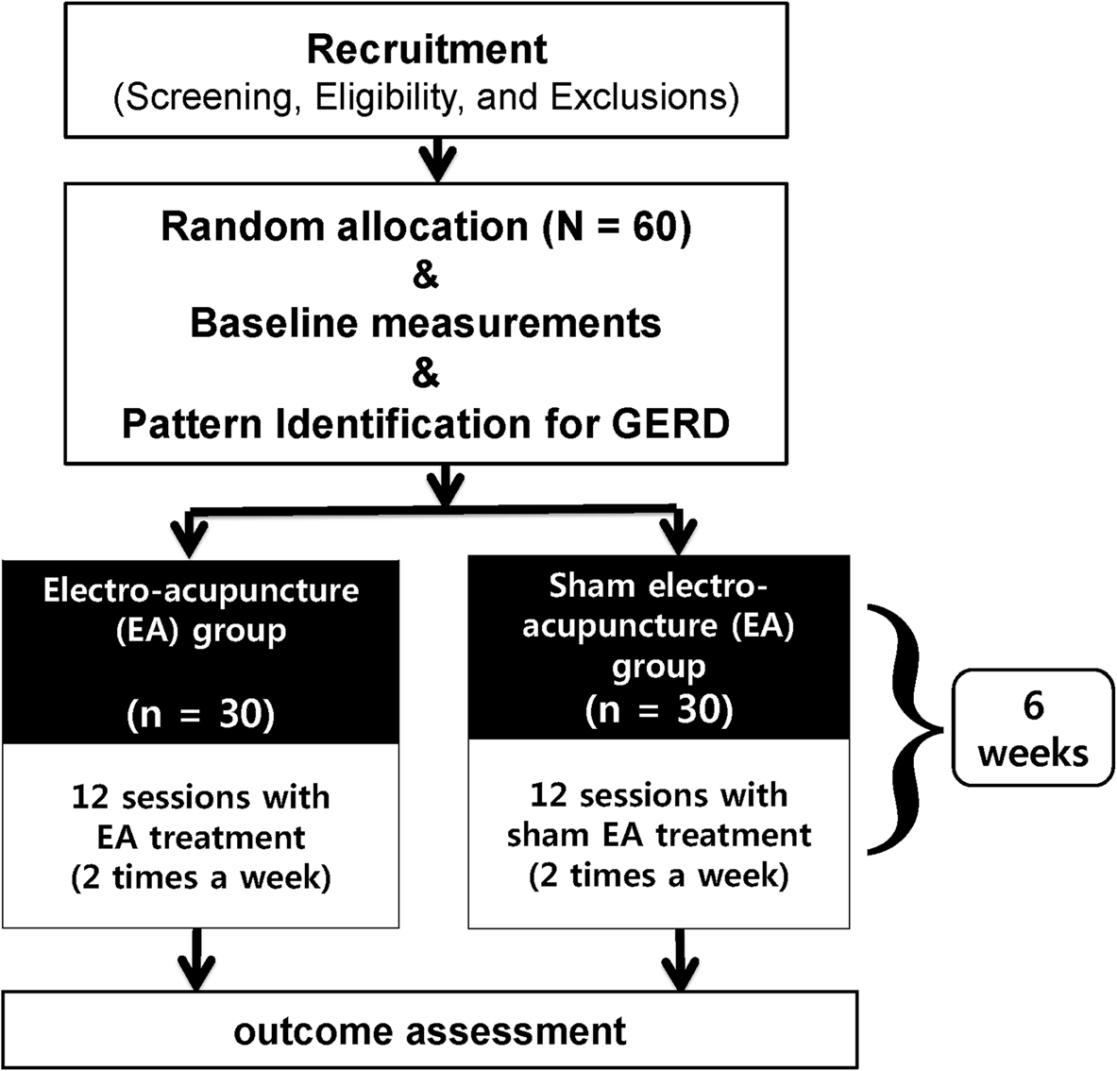
Timeline of the study

A single-center, parallel, randomized, patient-blinded, assessor-blinded, sham EA-controlled trial will be conducted at the Kyung Hee Korean Medicine Clinical Trial Center, which is affiliated with Kyung Hee University Korean Medicine Hospital in Seoul, Republic of Korea, from August 2015 to January 2016. Figure 1 shows the planned timeline of the trial.

### Inclusion and exclusion criteria

Participants will (1) be ages 19 to 75 years; (2) have been diagnosed with GERD (either erosive or nonerosive) no more than a year before the trial begins; (3) have had one or more of certain GERD symptoms in the month before the trial begins (heartburn, regurgitation, dyspepsia, epigastric pain, belching, nausea, globus (feeling of a lump in the esophagus), dysphagia, coughing, or hoarseness [18]); (4) have had these symptoms on more than 4 days (in the case of mild symptoms) or more than 2 days (in the case of moderate and severe symptoms) during the 7 days before the trial begins; (5) have continued to experience the symptoms stated above despite standard treatment for GERD—using either medication or lifestyle modification, or both—for 4 weeks during the year before the trial begins; and (6) agree to participate in this study, voluntarily signing a written informed consent form. Symptoms that are easily tolerated, with minimal discomfort not affecting normal activities, are defined as “mild”; those that are sufficient to affect normal activities are classified as “moderate,” and severe symptoms comprise those that markedly affect normal activities [19].

Participants will be excluded if they (1) have any current symptoms related to a structural disease that has been confirmed by endoscopy (e.g., gastrointestinal cancer, eosinophilic esophagitis, candida esophagitis, etc.); (2) have severe dysphagia, hematemesis, weight loss, or hematochezia; (3) have suffered from gastrointestinal cancer during the 5 years before the study begins; (4) have received chemotherapy for cancer during the month before the study begins; (5) have been diagnosed with ischemic heart disease (e.g., angina pectoris or myocardial infarction); (6) have an artificial cardiac pacemaker in the chest; (7) have experienced a hypersensitivity reaction after an acupuncture treatment, or show any other contraindications; (8) are taking part in any other clinical trial that could affect the results of this trial, or are being treated for GERD using any other Korean medical method (a minimum wash-out period of 2 weeks will be required for participation in this trial); (9) have difficulties attending the trial (e.g., serious mental illness, dementia, drug addiction, severe disorders in vision or hearing, illiteracy, time constraints, etc.), and (10) are pregnant or breastfeeding.

### Recruitment

We will advertise the study on the notice boards and the homepage of the hospital, as well as in local newspapers, and the investigators who are Korean medical doctors, will obtain informed consent from all participants. Specifically, two written informed consent forms (a general consent form and an additional consent provision for the collection and use of the participants’ biological material) will be given to the participants. After the participants sign the forms voluntarily, the participants will go through the screening test. We will provide a copy of the consent form and other related documentation to the participants upon request.

#### Randomization and allocation concealment

Sixty participants will be randomly assigned to either the EA or sham EA group. A random number table will be generated using block randomization with blocks of four by R for windows 3.2.1 (R Foundation for Statistical Computing, Vienna, Austria); to this end, the block randomization function will be used with blocks of four. The allocations will be kept sealed in a double-locked cabinet by an independent statistician. If the participants pass the screening test, they will be allocated according to the procedure above to conceal the allocation from both investigators and participants. After allocation, the statistician will email a unique random number and participant group allocation to both the clinical research coordinator and the Korean medicine doctors (KMDs). This feedback document will be kept in the master file for the trial.

### Blinding

In this study, the participants, the outcome assessors, and the data managers, including the statistician, will be blinded to the group allocation. The acupuncture practitioner will not be blinded because of the characteristics of the Park sham device. The investigators will not give any clues to the participants regarding group allocation, nor will the participants from one group meet those from the other group. To evaluate the success of blinding, a blinding index will be assessed after the participants’ final treatment. Blinding will be maintained until the study is finished. However, if a serious adverse event (AE) occurs, blinding will be lifted, and investigators will notify the participant involved of his/her group allocation in accordance with the standard operating procedures (SOPs).

### Intervention

In Table 1, we have provided detailed information regarding the EA treatment to be used; the treatment will be based on the revised Standards for Reporting Interventions in Clinical Trials of Acupuncture (STRICTA) [20]. Furthermore, we have tried to reconstruct a real clinical environment in the study design, using partially individualized EA treatments based on both meridian theory and the consensus of Korean medicine gastroenterologists. Individualized treatment will be carried out according to the participant’s pattern identification. At the first visit, the participant will respond to a GERD pattern identification tool [21], and the pattern of the participant will be identified as one of following four types: “liver qi invading the stomach,” “spleen-stomach weakness,” “spleen-stomach dampness-heat,” and “stomach yin deficiency.” The participants’ identified types will not change for the duration of the study.

**Table 1:** Acupuncture treatment details based on the STRICTA 2010 checklist

Item	Detail
1. Acupuncture rationale	1a) Style of acupuncture - EA based on traditional meridian theory.
	1b) Reasoning for treatment provided, based on historical context, literature sources, and/or consensus methods, with references where appropriate - Partially individualized manual EA treatments based on the traditional meridian theory, literature review, consensus by the experts in GERD and acupuncture, and clinical experience of Korean medicine gastroenterologist.
	1c) Extent to which treatment was varied - Partially individualized EA treatment, i.e., fixed points plus optional points according to pattern identification of the participant.
2. Details of needling	2a) Number of needle insertions per subject per session (mean and range where relevant) - From 18 to 20.
	2b) Names (or location if no standard name) of points used (uni/bilateral) - Eighteen fixed points: ST36, a point 0.5 cun below ST36, PC6, LI4, LR3, SP4, SP9, GB34 (bilateral), CV10, and CV13 (unilateral). - Optional points according to individual pattern identification (1) “Liver qi invading the stomach” type: SP11, LR2 (Rt. side) (2) “Spleen-stomach weakness” type: CV12 (one point), ST41 (Rt. side) (3) “Spleen-stomach dampness-heat” type: LR13, SP3 (Rt. side) (4) “Stomach yin deficiency” type: CV12 (one point), ST44 (Rt. side)
	2c) Depth of insertion, based on a specified unit of measurement, or on a particular tissue level - From 5 to 30 mm.
	2d) Response sought (e.g., *de qi* or muscle twitch response) - none
	2e) Needle stimulation (e.g., manual, electrical) - Electrical stimulation: 2/6 Hz mixed (ITO ES-160, Japan) - Acupoints with electrical stimulation: ST36, a point 0.5 cun below ST36, PC6, LI4 (bilateral), CV10, and CV13 (unilateral).
	2f) Needle retention time - 20 minutes.
	2g) Needle type (diameter, length, and manufacturer or material) - Park sham device with real and sham needle, i.e., sterilized stainless steel needle (0.25 x 40 mm, Dongbang Acupuncture Inc., Bundang, Sungnam, Korea).
3. Treatment regimen	3a) Number of treatment sessions - Twelve treatment sessions in both EA and sham EA groups.
	3b) Frequency and duration of treatment sessions - Twice weekly for 6 weeks, 20 minutes for each session.
4. Other components of treatment	4a) Details of other interventions administered to the acupuncture group (e.g., moxibustion, cupping, herbs, exercises, lifestyle advice) - Except for lifestyle modification and rescue medicine, other interventions during the study period will not be allowed.
	4b) Setting and context of treatment, including instructions to practitioners, and information and explanations to patients - Korean Medicine Clinical Trial Center in University hospitals - Participants will be informed about acupuncture treatment in the study as follows: “In this study, classic and nonclassic acupuncture treatment for GERD will be used based on traditional Korean medicine textbook and GERD- related literature.”
5. Practitioner background	5) Description of participating acupuncturists (qualification or professional affiliation, years in acupuncture practice, other relevant experience) - Two Korean Medicine Doctors who have licenses and at least 5 years of experience in gastrointestinal disorders. They went through training and simulation to ensure that they are able to provide identical EA treatment on the ground of predefined protocol and standard operating procedure.
6. Control or comparator interventions	6a) Rationale for the control or comparator in the context of the research question, with sources that justify this choice - A sham EA group using Park sham device with sham needle will be adopted.
	6b) Precise description of the control or comparator. If sham acupuncture or any other type of acupuncture-like control is used, provide details as for Items 1 to 3 above. - Participants in the sham EA group will receive acupuncture treatment after randomization twice weekly for 6 weeks. - Sham EA group: nonpenetrating, nonacupoint, nonelectric current (1) Device: Park sham device with sham needle (2) Acupoint: Eighteen acupoints. Each acupoint is 2 cm apart from the acupoints of EA group to the medial side. (The optional points in accordance with pattern identification will not be included) (3) Duration of treatment sessions: 20 minutes

*STRICTA* STandards for Reporting Interventions in Clinical Trials of Acupuncture; *EA* elctroacupuncture, *GERD*, gastroesophageal reflux disease, *ST* stomach, *PC* pericardium, *LI* large intestine, *LR* liver, *SP* spleen, *GB* gallbladder, *CV* conception vessel

The treatment will consist of 12 sessions across 6 weeks (two sessions each week); it will be performed by KMDs with more than 5 years of clinical experience in acupuncture, who will go through training and simulation to ensure identical acupuncture treatment in accordance with the SOPs. The minimum interval between treatment sessions will be 2 days. Lifestyle will be modified in both groups.

#### EA group

The number of inserted needles per participant in one session will range from 18 to 20. The basic points and optional points used will be included in the acupuncture treatment regimen. All participants will undergo acupuncture treatment at 18 fixed points. The details of the acupoints are shown in Table 1. Of these points, electrical stimulation will only be applied to ST36, a point 0.5 cm below ST36, PC6, LI4 (bilateral), CV10, and CV13 (unilateral). In addition, optional points will be selected on the basis of the participant’s pattern identification; the details are shown in Table 1.

We will use a Park sham device (Acuprime, Exeter, UK) with real penetrating needles [22]; the inserted needle of this device is a sterilized stainless steel needle (DongBang Acupucture Inc., Bundang, Sungnam, Republic of Korea) 40 mm in length and 0.25 mm in diameter. The needles will be inserted to a depth of between 5 mm and 30 mm, and they will remain in place for 20 minutes. In the case of EA points, the needles will be stimulated using a mixed electric current at a frequency of 2/6 Hz; to this end, the “fast plus slow” mode of the ES-160 (ITO Co., Ltd., Tokyo, Japan) will be used. The stimulation intensity will be adjusted depending on the degree of the participant’s tolerance.

#### Sham EA group

Participants in the sham EA group will be treated using 18 fixed acupoints. However, acupoints based on individual pattern identification will not be used. We will use a Park sham device with sham (blunted) needles [22] that do not penetrate the skin; this device has been described in previous studies [23–25]. At the EA points, the same accessories of the EA equipment will be assembled, and there will be a timer sound and a blinking light; however, no electrical current will be applied.

#### Lifestyle modification

In accordance with the clinical practice guidelines for GERD [26], as well as the SOP, lifestyle modification will be taught to participants by the KMD at every visit. Lifestyle modification will comprise (1) avoidance of foods that worsen GERD symptoms (e.g., coffee, alcohol, chocolate, fat, etc.), (2) avoidance of foods that cause heartburn (e.g., spicy food, fruit, carbonated drinks, etc.), (3) changes to lifestyle that decrease the exposure of the lower esophagus to gastric acid (e.g., weight loss, cessation of smoking and of drinking alcohol, raising the upper part of the bed, going to bed 2–3 hours after a meal) [27].

#### Distribution of rescue medicine

With regard to ethics, we will distribute the rescue medicine, named Norumo Dual Action Suspension (Il-Yang Pharmaceutical Co., Ltd., Gangnam, Seoul, Republic of Korea), to all participants. If participants experience intolerable GERD symptoms between visits, they will be permitted to take one pack. The ingredients of the medicine are sodium alginate, sodium bicarbonate, and calcium carbonate. The number of rescue medicines administered will be evaluated at each visit. If the number between visits is more than three, then the primary outcome, adequate relief (AR), will be classified as “no relief,” regardless of the participant’s answer.

### Withdrawal criteria

Participants will be withdrawn from the study if they (1) miss a scheduled EA treatment three times in a row or (2) take forbidden medication, herbal medicine, or undergo surgery related to GERD during the study period.

### Drugs forbidden by the study protocol

The following classes of drugs, all of which are used to treat symptoms of GERD, will not be allowed: PPIs, histamine receptor antagonists, prokinetics, mucosal protective drugs, antacids, anti-depressants, antianxiety drugs, lower esophageal sphincter agents, and herbal medicine.

### Outcome measurement

An independent assessor will give the participants a set of outcome measures; a schedule of these measures is summarized in Table 2.

**Table 2:** Study schedule for data collection

Measures	V1	V2	V3	V4	V5	V6	V7	V8	V9	V10	V11	V12	V13
Sociodemographic data	✔												
GERD pattern identification tool	✔												✔
Collection and distribution of diary	✔		✔		✔		✔		✔		✔		✔
Assessment of beliefs in the acupuncture effectiveness	✔												
Adequate relief (AR)		✔	✔	✔	✔	✔	✔	✔	✔	✔	✔	✔	✔
Nepean Dyspepsia Index - Korean (NDI-K)	✔					✔							✔
Numeric rating scale (NRS) for GERD	✔					✔							✔
Korean Gastrointestinal Symptom rating scale (KGSRS)	✔					✔							✔
EuroQol-5 Dimension (EQ-5D)	✔					✔							✔
Perceived Stress Scale-Korean (PSS)	✔					✔							✔
Gut hormones (gastrin and motilin)	✔												✔
Blood collection for cytokine analysis	✔												✔
Qi stagnation and spleen Qi deficiency questionnaire	✔												✔
PGIC GERD symptom						✔							✔
Blinding Index (BI) and cCredibility test													✔
Adverse events investigation		✔	✔	✔	✔	✔	✔	✔	✔	✔	✔	✔	✔
Assessment of concomitant therapy and lifestyle modification	✔	✔	✔	✔	✔	✔	✔	✔	✔	✔	✔	✔	✔
Evaluation of rescue medicines		✔	✔	✔	✔	✔	✔	✔	✔	✔	✔	✔	✔

#### Primary outcome

We set the “proportion of responders” (PR) [28, 29] as the primary outcome. PR is the proportion of participants who respond “yes” to more than half of the AR-related questions during the treatment period [30]. An assessor will ask the participants the following question at each visit during the treatment period (twice a week for 6 weeks): “Have you had adequate relief of the pain or discomfort caused to you by GERD since the last visit?” We will define AR as ‘improvement of GERD symptoms by ≥ 20 % since the last visit.

If participants report AR at more than half of all treatment sessions, they will be defined as responders, and we will compare the proportion of responders between the EA and sham EA groups.

#### Secondary outcomes

##### Nepean dyspepsia index - Korean version

The Nepean dyspepsia index (NDI), a disease-specific instrument used to assess GERD symptoms [31], is a reliable and valid questionnaire [32, 33]. In this trial, the Korean version of NDI (NDI-K) will be used. It was validated in 2004 by Lee et al. [34] and contains 40 items comprising two sections: symptom-based questions (duration, severity, and degree of 15 separate symptoms), and those pertaining to the quality of life (QoL). The patient will complete the questionnaire at baseline, visit 6, and visit 12.

##### Gastrointestinal hormone (gastrin, motilin)

GERD is related to weakness in the lower esophageal sphincter [35], and motilin [36] and gastrin [37, 38] have been reported to increase strength of the lower ESP (LESP). Motilin is an endogenous gastrointestinal hormone that increases upper GI tract motility, and it is considered an endogenous prokinetic hormone [39]. Gastrin is also a gastrointestinal polypeptide; it is thought to stimulate the lower esophageal sphincter by increasing the LESP [40]. However, controversy exists regarding whether EA increases gastrin and motilin levels [11, 13–16], so we will attempt to confirm the influence of EA on increases in motilin and gastrin in this trial. Specifically, a nurse will collect a 10-mL blood sample, in which the serum will be separated using a centrifuge. The sample will be analyzed using both a Human Motilin ELISA kit and a Human Gastrin ELISA kit (Mybiosource Inc., San Diego, California, USA). This will be carried out at baseline and visit 12.

##### Inflammatory cytokines

One report has claimed that cellular inflammatory processes are involved in the pathogenesis of GERD and its complications [17]. On this basis, we will evaluate serum IL-1, IL-6, IL-8, IL-4, and IL-10 (all inflammatory mediators) using the BD Facscalibur™ (BD Bioscience, CA, USA) at baseline and visit 12. IL-8 is expressed at high levels in the affected mucosae of patients with GERD [41–43]. IL-1, IL-6, IL-10, and IL-4 are also important mediators of the inflammatory responses related to GERD [17]. Through this analysis, we will clarify the influence of EA on the levels of cytokines, as well as the relation between these results and subjective symptoms.

##### EuroQol-5 Dimension

The EuroQol-5 Dimension (EQ-5D) [44] will be used to evaluate the participants’ QoL. The EQ-5D is a standardized preference-based questionnaire for measuring health-related QoL [30]; it consists of the EQ-5D index and the EQ-5D visual analog scale (VAS). The EQ-5D index is calculated on the basis of five dimensions: mobility, self-care, ability to undertake usual activities, pain, and anxiety/depression. The EQ-5D VAS measures the participant’s current health status using a standard vertical 20 cm VAS [45]. The top of this line is labeled “best imaginable health state” and the bottom “worst imaginable health state.” We will use a validated Korean version of EQ-5D [46] at baseline, visit 6, and visit 12.

##### Korean gastrointestinal symptom rating scale

The Korean gastrointestinal symptom rating scale (KGSRS) [47] will be used to evaluate the gastrointestinal discomfort of participants. This questionnaire consists of 16 items covering both upper and lower gastrointestinal symptoms. In this trial, we will evaluate changes in reflux, abdominal pain, and indigestion after treatment. The evaluation will be carried out at baseline, visit 6, and visit 12.

##### Eleven-point numerical rating scale

Participants will be asked to check the discomfort caused to them by GERD using an 11-point numerical rating scale. This will be performed at baseline, visit 6, and visit 12.

##### Perceived stress scale

The perceived stress scale (PSS) was developed and validated by Cohen et al. in 1983 [48]. We will use the PSS translated into Korean and validated by Lee [49]. This questionnaire evaluates a participant’s perceived stress during the last 1 month using a 5-point scale.

##### Blinding index and credibility assessment

Blinding of participants will be assessed using a blinding index (BI) [50]. Participants will answer the following question for each acupuncture point: “What type of acupuncture do you think you received?” The answer will be selected from three choices: “classic electroacupuncture,” “non-classic electroacupuncture,” or “don’t know.” In addition, the credibility of the treatment will be evaluated using the credibility assessment questionnaire. These evaluations will be conducted after EA treatment at visit 12.

##### Pattern identification for gastroesophageal reflux disease

The participants’ pattern will be classified using pattern identification for gastroesophageal reflux disease (PIGERD) [21]. Participants will be assigned EA treatment on the basis of the result of the PIGERD. This identification will be performed at visit 1 and visit 12.

##### The acupuncture beliefs scale

We will assess the participant’s belief in the efficacy of EA at visit 1 using the acupuncture beliefs scale [51], which consists of 36 items. Participants will respond on a 5-point Likert scale as follows: “strongly agree” (1 point), “agree” (2 points), “neutral” (3 points), “disagree” (4 points), and “strongly disagree” (5 points).

##### Patient global impression of change

The EA will be globally assessed by the participants using patient global impression of change—a self-reported seven-point categorical scale that is used to evaluate overall improvement after EA treatment [52]. Participants will evaluate improvements in their symptoms between baseline and visit 6, as well as between baseline and visit 12, by choosing one of the following answers: (1) very much improved, (2) much improved, (3) minimally improved, (4) no change, (5) minimally worse, (6) much worse, or (7) very much worse.

##### Qi-stagnation questionnaire

In traditional Korean medicine, the concept of qi stagnation is similar to that of stress in Western thinking [53]. The qi stagnation questionnaire is composed of 11 subjective items and one objective item; the items are used to diagnose qi stagnation. Using this questionnaire, we will evaluate the degree of qi stagnation in patients with GERD at visit 1 and visit 12.

##### Spleen qi deficiency questionnaire

The spleen qi deficiency questionnaire [54] will be used to diagnose whether the participant is in a state of spleen qi deficiency. This questionnaire consists of nine items concerning symptoms and two items regarding tongue and pulse diagnosis. The diagnosis will be performed at visit 1 and visit 12 by a KMD.

##### Evaluation of rescue medicine

We will quantify the amount of rescue medicines taken between visits.

### Sample size calculation

The required sample size was calculated on the basis of the difference in PR between the EA and sham EA groups. On the basis of previous research [55], we assumed the PR would be 92.8 % in the treatment group and 57.1 % in the control group. With a 5 % false positive error (*α* = 0.05, two-sided) and 80 % power (*ß* = 0.2) the required sample size in each group was *n* = 21.9. Assuming a 25 % of dropout rate, the real-world sample size will be *n* = 30 in each group, for a total of 60 participants.

### Statistical analysis

An independent statistician blinded to group allocation will carry out the statistical analysis using SPSS™ 18.0 (SPSS Inc., Chicago, Illinois, USA). All final data will be securely stored, and only the statistician will have access. We will perform both intention-to-treat (ITT) and per protocol (PP) analysis, with all analyses being based on the ITT principle. Any participant who receives EA more than nine times during the study period will be eligible for PP analysis.

For ITT analysis of secondary outcomes, we will use the “last observation carried forward” rule. In the case of the primary outcome, however, missing values will not be replaced, because of the characteristics of adequate relief (AR). We will only count actual responses regarding AR. When AR occurs in more than 50 % of the treatment sessions in which a study subject participated, the participant will be classified as a responder. All data will be shown as the mean ± standard deviation (SD) or as numbers and percentages. The statistical significance level will be set at 0.05 (two-sided), with 95 % confidence intervals (CI). The analysis for safety will also follow the principles of ITT.

#### Description of baseline characteristics and homogeneity of the two groups

To describe the baseline characteristics of continuous data, the mean ± SD, or range with minimum and maximum, will be used. In the case of dichotomous data, the frequency will be reported as a percentage. To evaluate the homogeneity of the baseline characteristics between the two groups, a two-sample t test for continuous data and the *χ*^2^ test for dichotomous data will be carried out. If there is a significant difference between the two groups in terms of baseline characteristics, we will use analysis of covariance (ANCOVA) or logistic regression.

#### Primary outcome

The primary outcome—PR after 6 weeks of EA—will be compared between the groups using the *χ*^2^ or Fisher’s exact test.

#### Secondary outcomes

The independent t test will be used to evaluate changes in the NDI-K score after 6 weeks of treatment. If there is a covariate at baseline, an ANCOVA will be performed. To compare variables between the two groups, the two-sample t test will be used for parametric analyses, and the Mann-Whitney U test, for nonparametric analyses.

A repeated measures analysis of variance or repeated measured ANCOVA will be used to analyze group-by-time interactions, as well as intergroup differences. The *χ*^2^ test or Fisher’s exact test will be used to analyze nominal variables in each group as well as to compare AEs between groups.

#### Data management

A well-trained assessor will collect data during the study period, and a blinded data manager will enter the data from the case report form into an electronic database. All data will be crosschecked and accessible only to the data manager, who is independent from the sponsor and competing interests.

### Safety

We will ask participants at each visit whether any AE has occurred or not. If any AEs occur, we will offer immediate and appropriate treatment to the participant and observe his/her progression through follow up. In addition, we will record the name, severity, duration, and cause of all AEs that occur during the period of study. Then, the investigator will assess causality between the AE and the intervention. If a correlation is noted, the investigator will take prompt and appropriate action. The causes of AEs during EA treatment will be reviewed by an independent monitor. If any serious AE occurs, we will report it to the Institutional Review Board (IRB) within 24 hours of recognition.

### Quality control

A well-trained clinical research associate independent from the investigators will regularly moderate the quality of this trial. The monitoring will consist of checking (1) informed consent and case report forms, (2) participants’ compliance with treatments, (3) the trial master file, (4) serious AEs, and (5) the data set.

### Ethical approval and registration

We will carry out this trial according to the standards of the International Committee on Harmonization of Good Clinical Practice and the revised version of the Declaration of Helsinki. An initial version of this protocol was approved by Kyung Hee University Korean Medicine Hospital IRB (KOMCIRB-150217-HRBR-009) on March 27, 2015.

If there is change in the protocol, the investigator will report this change to the relevant parties, such as the IRB, and trial participants. In addition, the investigators will be educated in maintaining the confidentiality of the participants’ personal information. This study is registered as a clinical trial at the Clinical Research Information Service (CRIS; KCT0001653; https://cris.nih.go.kr/cris/en/).

## Discussion

The current study will be a randomized, patient-blinded, assessor-blinded, sham-controlled trial to evaluate the effect of EA on GERD symptoms, as well as to clarify the influence of EA on esophageal motility-related variables. To our knowledge, insufficient evidence exists regarding the efficacy of EA in patients with GERD. Among the few studies that do exist on the topic, not one has used the Park sham device as a control. This trial is expected to clarify whether EA provides adequate symptom relief and improves QoL and whether GI hormone secretion and inflammatory cytokines are more affected by EA than by sham EA.

The Park device is the most commonly used placebo in acupuncture research [56], and it has been used as a control in many acupuncture RCTs [23–25, 57]. The device consists of a needle (either penetrating or blunted) and an additional plastic tube, and it has been validated in a previous study [58]; the two needle types are visually indistinguishable and have specific modalities. Therefore, we will use the Park device as a placebo control.

In this study, participants will only be included if they suffer GERD symptoms despite having undergone standard treatments, such as PPIs or lifestyle modification, before enrollment. In addition, we will allow the participants in both groups to modify their lifestyles as a part of conventional care. For this reason, we do not believe that withholding PPI treatment will raise an ethical problem. The manner of lifestyle modification varies; we have chosen a protocol from the Korean clinical practice guidelines because we would like to reflect the real setting of Korean clinical practice. In addition, we will check whether the lifestyle modifications have been well maintained.

One report addressing functional gastrointestinal disorders (FGIDs) recommended that participants be classified as either responders or nonresponders in terms of the primary outcome [28], whereas another study showed that a responder analysis may be an important addition to clinical trial reporting [59]. Still another [30] used PR as a primary outcome for evaluating the effect of acupuncture in functional dyspepsia, which is one of the FGIDs. Hence, we will also use PR as a primary outcome in this trial.

We have set the duration of EA at 6 weeks in the current study to reflect recommendations regarding treatment trials involving FGIDs. This study, therefore, will be able to test for an adequate duration of EA as well as to reflect clinical practice.

We will also evaluate the levels of gastrin, motilin, and inflammatory cytokines such as IL-1, IL-4, IL-6, and IL-8; all these are objective outcomes related GERD. In this way, we will elucidate the mechanism of GERD symptom improvement, as well as the correlation between objective and subjective, symptom-related outcomes.

This study has the following limitations. First, doctors will not be blinded to the group allocation because of the features of the Park device. Blinding implies keeping those who have an important role in the research, such as doctors who perform treatment, participants, outcome assessors, and data analysts, unaware of the treatment administered [60]. To overcome this limitation, the outcome assessor will be separated from the doctors, and the blinded assessor will measure and collect all outcome measures. Another limitation is that we will include participants without performing gastroscopy; rather, we will take a history from the participants. However, this may be regarded as pragmatic because it will more accurately reflect common clinical practice in Korean medicine.

This will be the first well-designed RCT using a validated device as a placebo control. The results will provide information about the efficacy and safety of EA as a treatment for GERD symptoms as well as evidence regarding the application of EA to treat GERD in real clinical practice.

### Trial status

We will recruit participants from August 2015. Interim analyses are not planned, and the primary results will be published by 2016.

## Additional file

Additional file 1: SPIRIT 2013 Checklist. Completed SPIRIT checklist. (PDF 57 kb)

## Abbreviations

GERD: gastroesophageal reflux disease
PPI: proton pump inhibitor
EA: electroacupuncture
ESP: esophageal sphincter pressure
TLESRs: transient lower esophageal sphincter relaxations
RCTs: randomized controlled trials
KMD: Korean Medicine Doctor
SOP: standard operating procedure
STRICTA: Standards for Reporting Interventions in Clinical Trials of Acupuncture
AR: adequate relief
PR: proportion of responders
NDI-K: Nepean dyspepsia index —Korean version
EQ-5D: European Quality of life-5 Dimension
PSS: perceived stress scale
ANCOVA: analysis of covariance
AEs: adverse events
IRB: Institutional Review Board
FGIDs: functional gastrointestinal disorders
